# PICK1 Deficiency Exacerbates Sepsis-Associated Acute Kidney Injury

**DOI:** 10.1155/2021/9884297

**Published:** 2021-07-08

**Authors:** Qian Dou, Hang Tong, Yichun Yang, Han Zhang, Hua Gan

**Affiliations:** ^1^Department of Nephrology, The First Affiliated Hospital of Chongqing Medical University, Chongqing 400016, China; ^2^Department of Urology, The First Affiliated Hospital of Chongqing Medical University, Chongqing 400016, China; ^3^Department of Gastroenterology, The First Affiliated Hospital of Chongqing Medical University, Chongqing 400016, China

## Abstract

We performed in vitro and in vivo experiments to explore the role of protein kinase C-binding protein 1 (PICK1), an intracellular transporter involved in oxidative stress-related neuronal diseases, in sepsis-related acute kidney injury (AKI). Firstly, PCR, western blotting, and immunohistochemistry were used to observe the expression of PICK1 after lipopolysaccharide- (LPS-) induced AKI. Secondly, by inhibiting PICK1 in vivo and silencing PICK1 in vitro, we further explored the effect of PICK1 on AKI. Finally, the relationship between PICK1 and oxidative stress and the related mechanisms were explored. We found that the expression of PICK1 was increased in LPS-induced AKI models both in vitro and in vivo. PICK1 silencing significantly aggravated LPS-induced apoptosis, accompanied by ROS production in renal tubular epithelial cells. FSC231, a PICK1-specific inhibitor, aggravated LPS-induced kidney injury. Besides, NAC (N-acetylcysteine), a potent ROS scavenger, significantly inhibited the PICK1-silencing-induced apoptosis. In conclusion, PICK1 might protect renal tubular epithelial cells from LPS-induced apoptosis by reducing excessive ROS, making PICK1 a promising preventive target in LPS-induced AKI.

## 1. Introduction

Acute kidney injury (AKI) is common in hospitalized patients, with various complications and high mortality, and brings a great challenge for clinicians [1–3]. Sepsis is the most common cause of AKI in critically ill patients, [4] and lipopolysaccharide (LPS) is an important initiating factor of sepsis, which can induce cytokine storm, oxidative stress, hypotension, insufficient renal perfusion, premature senescence, and eventually lead to the gradual decline of renal function, [5, 6] thus participating in the occurrence and development of sepsis-associated acute kidney injury (SA-AKI). LPS would make a valuable contribution towards the pathogenesis of sepsis-associated AKI. Moreover, apoptosis of renal tubular epithelial cells (TECs) also contributes to the progress of AKI [7–9]. Apoptosis induced by endotoxin plays an important role in sepsis-associated acute renal injury. Therefore, it is necessary to understand the potential mechanism of LPS-induced renal tubular cell injury and apoptosis.

Reactive oxygen species (ROS), which contained exogenous oxidants and active oxygenated compounds produced in oxidative metabolism in vivo, plays an essential role in cell apoptosis [10, 11]. When cells are exposed to harmful stimulation, a lot of ROS will be produced, leading to the oxidant-antioxidant imbalance [12–14]. Recent studies have shown that oxidative stress is the leading cause of LPS-induced mitochondrial damage in renal cells [15], and the mitochondrial dysfunction in turn triggers apoptosis by activating the cascade of tandem proteases [16–19]. Therefore, reducing oxidative stress may be an effective way to decrease LPS-induced apoptosis of TECs.

Protein interacting with C-kinase 1 (PICK1) is a unique protein containing both BAR (Bin/Amphiphysin/Rvs) and PDZ (PSD-95/DlgA/ZO-1) domains. This particular structure enables PICK1 to bind to various membrane proteins, thus initiating multiple functions [20–22]. PICK1 is widely expressed in various organs and has been found to participate in the oxidative regulation by regulating glutathione (GSH, an important antioxidant) [23] in the lung [24] and liver [25]. However, the research on the relationship between PICK1 and SA-AKI is relatively rare.

In our study, using a septic mouse model by injecting LPS, we investigated the role of PICK1 in the pathophysiological process of septic-induced AKI and explored the underlying mechanism.

## 2. Materials and Methods

### 2.1. Animal Models and Groups

Our animal experiment was carried out in the Animal Laboratory Center of Chongqing Medical University (Chongqing, China). Male C57BL/6 mice (6–8 weeks old, 20–25 g) purchased from the Animal Laboratory Center of The First Affiliated Hospital of Chongqing Medical University were used in this study. The Animal Experimentation Ethics Committee of The First Affiliated Hospital of Chongqing Medical University approved all animal experiments. Mice were housed under specific pathogen-free conditions, and the standard rodent chow and water were available ad libitum. The septic AKI animal model was established by intraperitoneal injection of 10 mg/kg of LPS for 24 hours [15, 26].

FSC231 (529531, Merck, Germany), a specific PICK1 inhibitor [27], was given to examine the role of PICK1 in LPS-induced AKI. C57BL/6 mice were randomly divided into three groups (*n* = 6 in each group): the control group, LPS group, and LPS+FSC231 group. The LPS group was given intraperitoneal injection of LPS, and the control group was given the same dose of saline. The LPS+FSC231 group was injected with FSC231 (78.4 *μ*g/g) for five consecutive days, followed by LPS administration 2 hours after the last dose of FSC231 [25]. Renal tissue and blood samples were collected after 24 hours of LPS treatment.

### 2.2. Assessment of Kidney Function

Blood samples were collected from the eyeballs. According to the manufacturer's illustration, blood urea nitrogen (BUN) and serum creatinine (Scr) were measured enzymatically.

### 2.3. Histology Analyses

Kidney specimens were fixed with 4% paraformaldehyde, embedded in paraffin, sectioned into 4 *μ*m thick, and stained with hematoxylin and eosin. The pathological changes of renal tissue were observed under the light microscope. A semiquantitative scoring method was used to evaluate the degree of renal tubular injury: 0 for less than 5%, 1 for 5%-25%, 2 for 25%-50%, 3 for 50%-75%, and 4 for more than 75%. Ten areas in each section were randomly observed under the light microscope (400x magnification) to evaluate tubular epithelial necrosis, lumen dilatation, and tubular injury.

### 2.4. Immunohistochemistry

The expression of PICK1 and cleaved caspase 3 were detected by immunohistochemistry. The dewaxed slices were put into citrate buffer (0.01 M, pH 6.0) and heated in microwave oven for 20 minutes. Then, we complete the remaining dyeing steps according to the manufacturer's immunoassay kit instructions (ZSGB-BIO Technology Co., Ltd., Beijing, China). PICK1 (10983-2-AP, Proteintech, China) and cleaved caspase 3 (19677-1-AP, Proteintech, China) were used as the primary antibody at a dilution of 1 : 200. The images were taken with a digital camera under a microscope.

### 2.5. Cell Culture and Treatment

The human renal proximal tubular epithelial cell line (HK2) was presented by Dr. Jiang, Center for kidney disease, Chongqing Medical University. The cells were cultured in 1640 medium (Gibco, USA) containing 10% FBS (PAN-Biotech, Adenbach, Germany) and 1% penicillin-streptomycin (Beyotime, China) at 37°C in 5% CO_2_ wet air with or without Lenti-NC and Lenti-PICK1 to silence the expression of PICK1 (NC and sh-PICK1). And then, for the treatment with LPS (15 *μ*g/ml) for 24 hours, the cells were divided into the WT group, WT+LPS group, NC+LPS group, and sh-PICK1+LPS group for further experiments.

### 2.6. Transfection

Lenti-NC and Lenti-PICK1 were built at Shanghai Genechem Co., Ltd. (Shanghai, China). HK2 cells were seeded into 6-well plates. When the cell fusion reached 50%, the medium containing lentivirus and HitransG A was added. After 24 hours of incubation, the medium was changed, and the cells were cultured for another 24 hours for further analysis. Besides, stably transfected cell lines were screened by puromycin (2 ng/ml). Then, cells were cultured with the optimum concentration of LPS for the given time.

### 2.7. Immunofluorescence Staining

HK2 were inoculated in 24 aperture plates after the cells reached 40% to 50% confluency, treated with LPS for 24 hours after adhering the wall, fixed with 4% paraformaldehyde for 15 minutes at room temperature, and then immersed with 0.1% TritonX-100 (p0096, Jiangsu, China) and 5% goat serum for 15 min and 30 min, respectively. After culture with PICK1 antibody (1 : 200, Proteintech, China) at 4°C overnight, goat anti-rabbit IgG (1 : 20, Proteintech, China) was added in the dark for 1 hour. The cells were then stained with 2-(4-aminophenyl)-6-indoleamine dihydrochloride (DAPI) for 10 minutes. Zeiss LSM 800 confocal laser scanning microscope (Carl Zeiss, Germany) was used for image acquisition and observation.

### 2.8. GSH Measurement

According to the manufacturer's illustration, GSH-Px (Total Glutathione Peroxidase Test Kit, S0053, Beyotime, China) was determined with the corresponding test kit.

### 2.9. Detection of Intracellular ROS Levels

According to the manufacturer's illustration, ROS was determined with the corresponding test kit (Reactive Oxygen Species Assay Kit, S0053, Beyotime, China). In short, the treated cells were incubated with DCFH-DA (10 *μ*mol/l) in the dark at 37°C for 30 minutes and then washed with serum-free medium. The fluorescence was observed under flow cytometry. The excitation and emission wavelengths were 488 nm and 525 nm, respectively.

### 2.10. TUNEL Staining

According to the manufacturer's instructions, the apoptotic cells in renal tissue were evaluated with the TACS TdT In Situ Apoptosis Detection kit (Roche, Germany). Renal tissue sections were deparaffinized and hydrated as per the standard procedure and digested with proteinase K solution at 37°C for 30 min. After the endogenous peroxidase activity was quenched, the sections were incubated with TdT labeling reaction mixture at 37°C for one hour and then treated with streptavidin horseradish peroxidase. Subsequently, the prepared samples were washed with phosphate buffer for colorimetric analysis. An optical microscope was used to analyze the results.

### 2.11. Hoechst 33258 Staining

According to the manufacturer's instructions, the cells were washed twice with PBS. Then, the samples were fixed with 4% formaldehyde for 15 min at room temperature and stained with Hoechst 33258 (Beyotime, China) solution in the dark for 5 minutes. After incubation, the apoptotic cells were observed under a fluorescence microscope.

### 2.12. Real-Time Quantitative Polymerase Chain Reaction (RT-PCR)

According to the manufacturer's instructions, cell RNA was extracted with TRIzol reagent (Invitrogen, Shanghai, China). Total RNA was reverse transcribed into cDNA by Prime Script RT Kit (Takara Biotechnology Inc., Shiga, Japan). RT-qPCR was performed with the following primer pairs:

PICK1-F 5′-AGTTCGGCATTCGGCTTC-3′, PICK1-R 5′-GAAGCCGAATGCCGAACT-3′, GAPDH-F 5′-GGTGAAGGTCGGAGTCAACG-3′, and GAPDH-R 5′-CAAAGTTGTCATGGATGHACC-3′.

The target gene level was normalized with GAPDH level, and the mRNA level was calculated by the standard method.

### 2.13. Western Blot

RIPA buffer containing PMSF and phosphatase inhibitors was used to extract the proteins of renal tissue and HK2 cells protein (all from Beyotime, China). According to the manufacturer's protocol, the protein concentration was determined by the BCA protein assay kit. The protein samples of each group were separated by SDS-PAGE and transferred to PVDF membranes (MILI Bloomberg, Massachusetts). The PVDF membranes were sealed with rapid blocking solution at room temperature. The primary antibodies included rabbit anti PICK1 (1 : 500, protein tech,) rabbit anti Cleaved Caspase-3, rabbit anti Bax, and rabbit anti BCL-2 (1 : 500, Proteintech, China), rabbit anti ASK1 (1 : 500,Proteintech, China), rabbit anti p38MAPK (1 : 500, Proteintech, China), and rabbit anti GAPDH (1 : 8000; Proteintech, China). Using the HRP-labeled secondary antibody (1 : 3000 dilution) to incubate the membrane at 37°C for 1 hour. Finally, the fusion imaging system is used to detect the relative densities of the bands.

### 2.14. Flow Cytometry

Annexin V-FITC/PC was used to detect apoptosis with the test kit (Beyotime, China) according to the manufacturer's instructions. The apoptosis was analyzed by flow cytometry (BD Biosciences, USA).

### 2.15. CCK-8

According to the manufacturer's illustration, the proliferation of pretreatment cell samples was detected by the CCK-8 kit (C0038, Beyotime, China). We add 10 *μ*l reagent to each sample for two hours and then detect the absorbance at 450 nm wavelength.

### 2.16. Statistical Analysis

All data were expressed as the means ± standard deviation and analyzed by GraphPad Prism 9.0 software using one-way ANOVA or *t*-test. Differences were considered significant at *P* < 0.05.

## 3. Results

### 3.1. PICK1 Was Increased in the LPS-Induced Renal Proximal Tubular Cells

**Figure 1:**
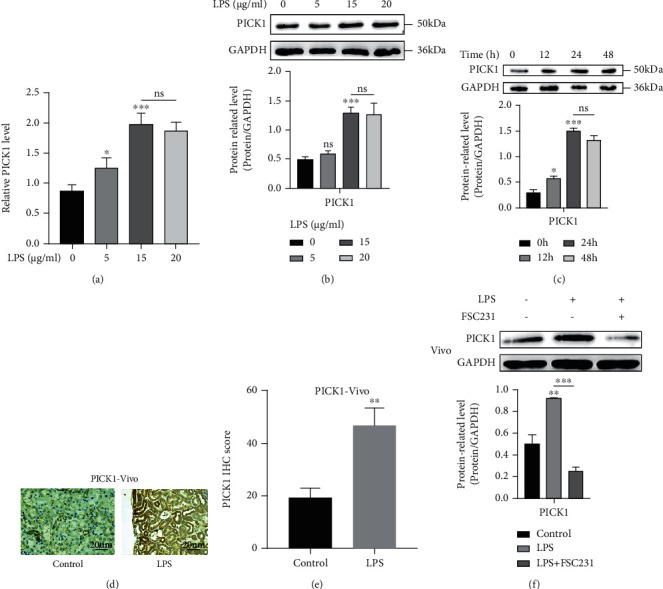
PICK1 was increased in LPS-induced AKI models both in vitro and in vivo. (a, b) PCR and western blotting results after treatment with different concentrations of LPS for 24 hours (0 *μ*g/ml versus 5 *μ*g/ml, 0 *μ*g/ml versus 15 *μ*g/ml; ^∗^*P* < 0.05, ^∗∗∗^*P* < 0.001). (c) Western blotting results after LPS treatment for different times (0 h versus 12 h, 0 h versus 24 h; ^∗^*P* < 0.05, ^∗∗∗^*P* < 0.001). (d, e) Representative immunohistochemical images of pretreated renal tissue (magnification ×400, scale = 20 *μ*m, the control versus LPS, ^∗∗^*P* < 0.01). (f) Representative western blotting analysis showed the expression of PICK1 in the kidney of mice treated with LPS (10 mg/kg) and FSC231 (78.4 *μ*g/g) (the control versus LPS, ^∗∗^*P* < 0.01).

Western blotting and qPCR were used to investigate the effect of LPS on the expression of PICK1 in vitro. We found that the expression of PICK1 increased after LPS treatment at different concentrations (0, 5, 15, and 20 *μ*g/ml, Figures 1(a) and 1(b)). In HK2 cells, PICK1 gradually increased with time after LPS treatment (15 *μ*g/ml, Figure 1(c)). During our observation period, the expression of PICK1 reached the peak after treatment with 15 *μ*g/ml LPS for 24 hours. Therefore, LPS induced the increasing expression of PICK1 protein in a time- and dose-dependent manner.

The level of PICK1 in LPS-treated mouse kidney was also detected in vivo. After treatment with LPS (10 mg/kg) for 24 h, the expression of PICK1 in the renal cortex and medulla significantly increased as showed in the immunohistochemical staining image (Figures 1(d) and 1(e)). The bands of western blotting also showed that PICK1 in the kidney tissue of LPS-induced AKI mice was increased, and FSC231 decreased the level of PICK1 (Figure 1(f)).

### 3.2. Inhibition of PICK1 Aggravated the LPS-Induced AKI

**Figure 2:**
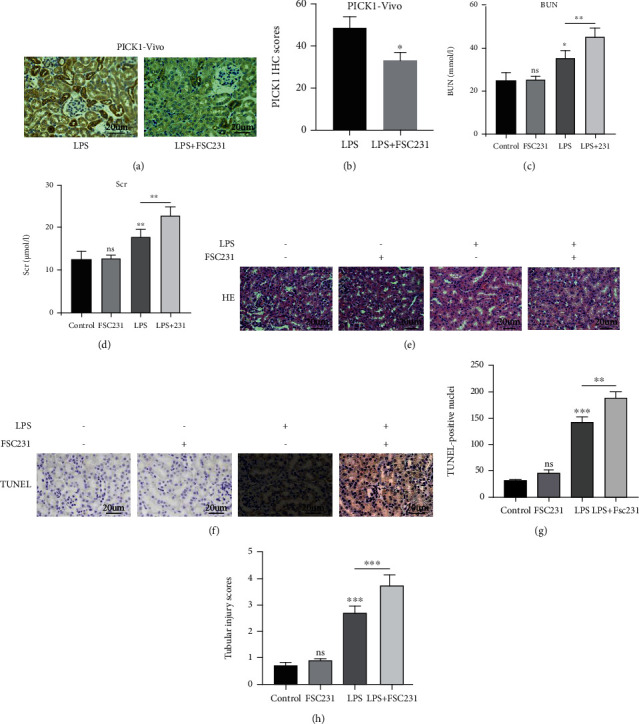
PICK1 inhibition aggravated LPS-induced AKI. (a, b) The results of immunohistochemistry (magnification ×400, scale = 20 *μ*m, LPS versus LPS+FSC231, ^∗^*P* < 0.05). (c) The results of BUN (the control versus LPS, LPS versus LPS+FSC231; ^∗∗∗^*P* < 0.001). (d) The results of Scr (the control versus LPS, LPS versus LPS+FSC231, ^∗∗^*P* < 0.01). (e) Hematoxylin-eosin (H & E) staining showed the renal histological damage (magnification ×400, scale = 20 *μ*m). (f, g) Representative images and statistical analysis of TUNEL-positive cells (magnification ×400, scale = 20 *μ*m, the control versus LPS, LPS versus LPS+FSC231; ^∗∗^P < 0.01, ^∗∗∗^*P* < 0.001). (h) The results of tubular injury scores (the control versus LPS, LPS versus LPS+FSC231, ^∗∗∗^*P* < 0.001).

FSC231, acting as a particular PICK1 inhibitor [27], was administered to investigate the role of PICK1 in LPS-induced AKI. The immunohistochemistry results showed that FSC231 reduced the PICK1 expression in LPS-induced AKI (Figures 2(a) and 2(b)). Both BUN and serum creatinine (Scr) presented a rapid increase after FSC231 treatment (Figures 2(c) and 2(d)). Histological analysis revealed that the expression of PICK1 in the kidney tissue of LPS-induced AKI mice was increased, and FSC231 treatment aggravated AKI, which was reflected by the exfoliated TECs, the damaged tubular structure, and the necrotic epithelial cells (Figures 2(e) and 2(h)). The number of TUNEL-positive cells in the kidney of mice pretreated with PICK1 inhibitor FSC231 was also increased (Figures 2(f) and 2(g)). Therefore, the inhibition of PICK1 aggravated the LPS-induced AKI in vivo. However, the appeal results all show that using FSC231 alone without LPS have no significant effect on tissue morphology and function.

### 3.3. PICK1 Silencing Promoted TEC Apoptosis

**Figure 3:**
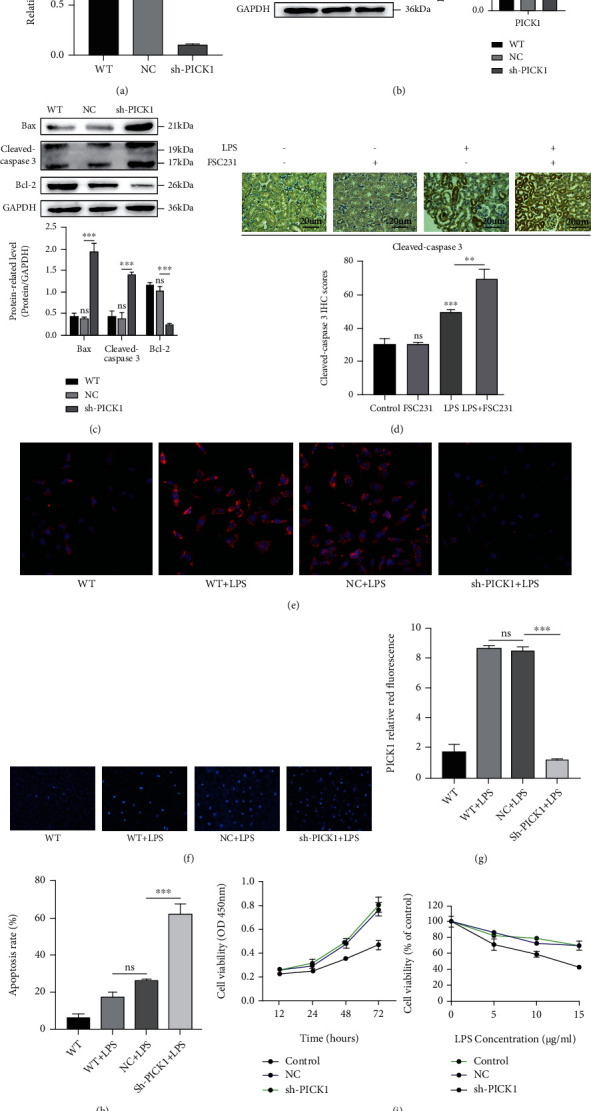
Downregulation of PICK1 aggravated LPS-induced apoptosis of HK2 cells. (a, b) The levels of PICK1 mRNA and protein were detected by qPCR and WB (NC versus sh-PICK1, ^∗∗^*P* < 0.01). (c) The expression levels of apoptosis-related proteins Bcl-2, Bax, and cleaved caspase 3 were detected by western blotting (NC versus sh-PICK1, ^∗∗∗^*P* < 0.001). (d) Representative cleaved caspase 3 level detected by immunohistochemistry (the control versus LPS, LPS versus LPS+FSC231; ^∗∗^*P* < 0.01, ^∗∗∗^*P* < 0.001). (e, g) Representative immunofluorescence results (WT+LPS versus NC+LPS, NC+LPS versus sh-PICK1+LPS; ^∗∗∗^*P* < 0.001). (f–h) The apoptosis of HK2 cells was detected by Hoechst 33258 staining (magnification ×200, scale = 50 *μ*m, WT+LPS versus NC+LPS, NC+LPS versus sh-PICK1+LPS; ^∗∗∗^*P* < 0.001). (f, h) Representative Hoechst 33258 staining results (WT+LPS versus NC+LPS, NC+LPS versus sh-PICK1+LPS; ^∗∗∗^*P* < 0.001). (i) The apoptosis of HK2 cells was detected by CCK-8.

Compared with the control group, after 24 hours of transfecting, PICK1 shRNA significantly reduced both the mRNA and protein of PICK1 (Figures 3(a) and 3(b)). The proapoptotic protein Bax and cleaved-caspase 3 was upregulated while the antiapoptotic protein Bcl-2 was downregulated after PICK1 silencing (Figure 3(c)). Similarly, the expression of cleaved-caspase 3 in the LPS+FSC231 group was significantly increased in immunohistochemistry, but in the case of using FSC231 alone, the expression of cleaved-caspase 3 did not change significantly (Figure 3(d)), suggesting that under the conditions of LPS-induced septic kidney injury, PICK1 inhibition aggravated the apoptosis of renal cells. The immunofluorescence results of HK2 cells showed that LPS could increase the expression of PICK1, but shRNA could decrease PICK1 (Figures 3(e) and 3(g)). Hoechst 33258 staining showed that PICK1 silencing evidently exacerbated LPS-induced apoptosis (Figures 3(f) and 3(h)). Moreover, the CCK-8 test showed that the proliferation of cells in the PICK1 knockdown group was significantly slowed down at different times and concentrations (Figure 3(i)). These results indicated that PICK1 was involved in the proapoptotic effect of LPS on HK2 cells.

### 3.4. PICK1 Inhibition Increased the Production of Peroxide and Activated the ASK1-p38 Apoptotic Pathway after LPS Treatment

**Figure 4:**
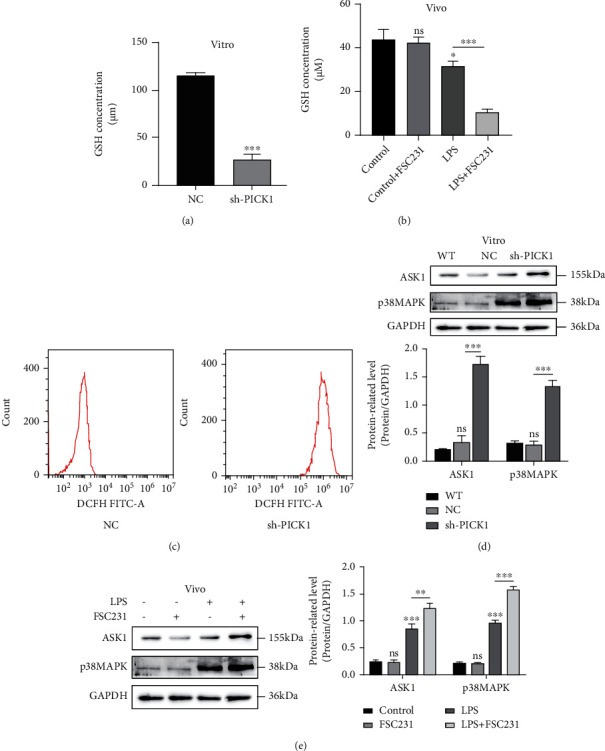
Knockdown of PICK1 increased the production of peroxide and activated the apoptotic pathway after LPS treatment. (a) After treatment with LPS for 24 hours, the content of GSH in the NC and sh-PICK1 groups (NC versus sh-PICK1, ^∗∗∗^*P* < 0.001). (b) The content of GSH in each group of mouse model (LPS versus LPS+FSC231, ^∗∗∗^*P* < 0.001). (c) Representative fluorescence images of ROS. (d, e) Representative western images of the ASK1 and p38MAPK pathway (NC versus sh-PICK1, ^∗∗^*P* < 0.01; ASK1: the control versus LPS, ^∗∗∗^*P* < 0.001; LPS versus LPS+FSC231, ^∗∗^*P* < 0.01; p38MAPK: the control versus LPS, ^∗∗∗^*P* < 0.001; LPS versus LPS+FSC231, ^∗∗∗^*P* < 0.001).

As an important intracellular regulatory metabolite, GSH could act as a direct antioxidant to participate in biotransformation. We detected the changes in GSH and ROS content after LPS pretreatment. GSH measurement result showed that the content of GSH in the PICK1 inhibition group was significantly lower than that in the NC group both in vivo and in vitro (Figures 4(a) and 4(b)). The results of flow cytometry indicated that the peroxide product in the sh-PICK1 group was significantly higher than that in the NC group (Figure 4(c)). Previous studies have shown that ROS can initiate apoptosis by activating the ASK1-p38MAPK signaling pathway [44]. Our western blotting showed that PICK1 inhibition upregulated the expression of ASK1 and p38MAPK both in the HK2 and mouse model, suggesting that PICK1 inhibition could activate the ASK1-p38MAPK signaling pathway (Figures 4(d) and 4(e)). Therefore, PICK1 silencing might be involved in LPS-induced ROS production and the activation of the ASK1-p38MAPK signaling pathway in vitro.

### 3.5. NAC Inhibited the LPS-Induced Apoptosis and ROS

**Figure 5:**
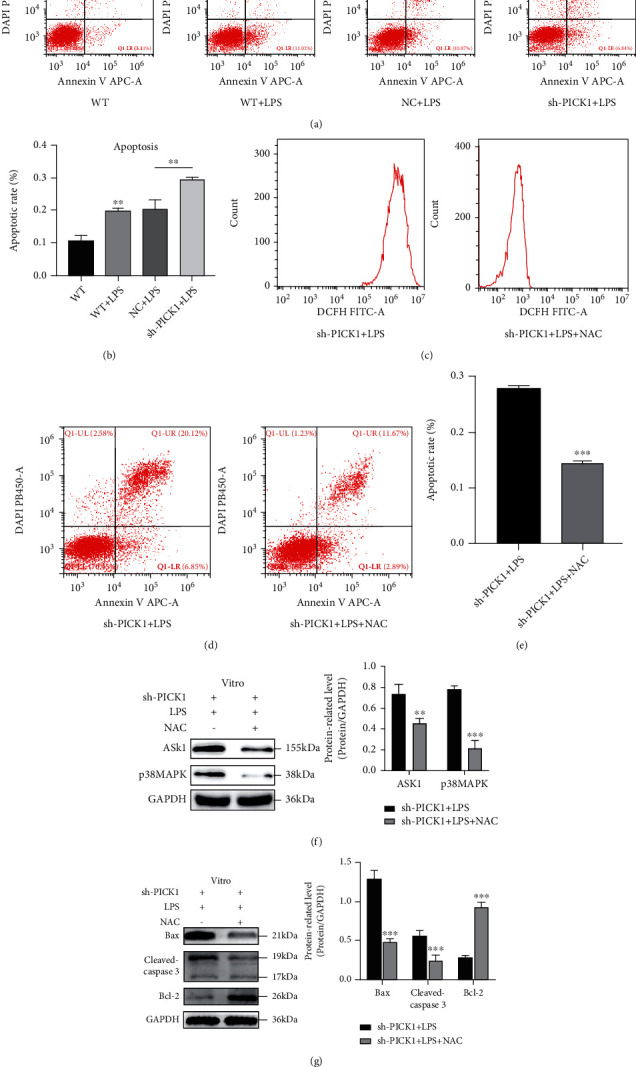
Inhibition of PICK1 expression could increase LPS-induced apoptosis, and NAC could alleviate pretreatment-induced apoptosis. (a, b) After 24 hours of LPS treatment in the different groups, apoptosis rate was detected by flow cytometry (WT versus WT+LPS, NC+LPS versus sh-PICK1+LPS; ^∗∗^*P* < 0.01). (c–e) HK2 cells were pretreated with 10 mM NAC for 1 h and then treated with LPS for another 24 h. Representative images of ROS and apoptosis rate in flow cytometry after NAC and LPS treatment (sh-PICK1+LPS versus sh-PICK1+LPS+NAC, ^∗∗∗^*P* < 0.001). (f) After treatment with NAC, the ASK1 and p38MAPK were detected by western blotting (sh-PICK1+LPS versus sh-PICK1+LPS+NAC; ^∗∗^*P* < 0.01, ^∗∗∗^*P* < 0.001). (f) After treatment with NAC, the apoptotic protein was detected by western blotting (sh-PICK1+LPS versus sh-PICK1+LPS+NAC, ^∗∗∗^*P* < 0.001).

NAC is an important scavenger for oxidation products in vivo [15]. We further explored the role of NAC on LPS-induced apoptosis in TECs. Flow cytometry showed that PICK1 silencing exacerbated LPS-induced early apoptosis (Figures 5(a) and 5(b)). Additional pretreatment with 10 mM NAC for one hour significantly downregulated the PICK1-silencing-exacerbated ROS and TEC apoptosis (Figure 5(c)–5(e)). At the same time, the expression of ASK1 and p38MAPK was decreased, and the proapoptotic protein Bax and cleaved-caspase 3 was downregulated while the antiapoptotic protein Bcl-2 was upregulated after NAC pretreatment detected by western blotting (Figures 5(f) and 5(g)). Based on our results, ROS-mediated apoptosis may participate in PICK1-inhibition-induced HK2 cell apoptosis through activating the ASK1-p38MAPK signaling pathway.

## 4. Discussion

Acute kidney injury (AKI) is a significant challenge for clinicians, and sepsis-associated acute kidney injury (SA-AKI) has been proved to increase the mortality of children and adults [1–3]. Endotoxin, especially lipopolysaccharide (LPS), is a common cause of SA-AKI [5]. However, since the lack of understanding of the complex pathophysiological mechanism of LPS-induced AKI, there is no effective intervention so far. PICK1 has been reported to have an antioxidant effect in the nervous system [23], but the role of PICK1 in AKI has barely been reported. Thus, we investigated whether PICK1 played a vital role in LPS-induced AKI in vivo and in vitro and found that PICK1 deficiency exacerbated SA-AKI.

Apoptosis is a programmed cell death necessary for cellular homeostasis [28]; however, excessive apoptosis can also cause tissue damage [29, 30]. The regulation of apoptosis involves many steps, and there are 3 main apoptosis regulating pathways as follows: the exogenous pathway, in which the death receptor pathway is a very important part [31]; in the intrinsic pathway, in which the mitochondrial apoptotic signaling pathway plays a pivotal role [32]; and in the endoplasmic reticulum stress-induced pathway, in which ROS destroyed the ER function and initiated unfolded protein response (UPR) and endoplasmic reticulum stress (ERS) in vivo and vitro, which might be an important mechanism that lead to tissue damage and cell apoptosis [33]. This study found that PICK1 inhibitor FSC231 significantly promoted the apoptosis of renal tubular epithelial cells in vivo and aggravated the LPS-induced renal injury. However, the use of FSC231 alone has no obvious effect on the functions of the kidney tissue. Therefore, PICK1 has a protective effect on AKI induced by LPS.

Oxidative stress is an inevitable reaction in life [34]. Various harmful stimuli can break the balance of oxidative stress, leading to cell apoptosis and pathological damage [12–14]. Previous studies have shown that oxidative stress played a vital role in the pathogenesis of septic kidney injury [15]. Oxidative stress and its induced apoptosis were confirmed to be essential pathogenic factors in chronic renal failure (CRF), [35] renal ischemia-reperfusion injury(IR) [36], acute renal injury (AKI) [37], and diabetic nephropathy (DN) [38]. Among various mechanisms, the endogenous pathway of mitochondrial initiation; the regulation of the Bcl-2 family, NF-*κ*B, and MAPK family; and the activation of caspase were most closely related to oxidative stress-induced apoptosis [39]. In our septic AKI model, we found that oxidative stress was the main factor leading to mitochondrial dysfunction, which caused a large-scale production of reactive oxygen species (ROS). JNK and p38MAPK, members of the MAPKs family, could transmit apoptosis signal to the mitochondria, release cytochrome C, activate specific caspase enzyme, and induce apoptosis.

Previous studies have shown that PICK1 interacts with various neurotransmitter receptors, enzymes, and transporters through its unique structure to affect synaptic function, leading to nerve damage, such as epilepsy and Parkinson's disease [22, 23, 40]. PICK1 was also involved in breast cancer by inhibiting TGF-*β* signaling, thus initiating early cancer [41]. Besides, PICK1 deficiency aggravated sepsis-induced acute lung injury through lysosomal injury [24, 42]. And PICK1 may mediate the prosurvival activity of PKCalpha by serving as a molecular link between PKCalpha and the mitochondria, which results in a more stable mitochondrial membrane potential, enhances phosphorylation of the anti-apoptotic Bcl-2 protein, and decreases dimerization of the.

proapoptotic Bax protein [45]. In other words, PICK1 was involved in the pathophysiological changes of many diseases. Recently, PICK1 has been reported to regulate glutathione (GSH) homeostasis, indicating that PICK1 may play a key role in oxidative stress [23]. However, the relationship between PICK1 and LPS-induced AKI remains unclear.

In our research, PICK1 acted as a negative regulator of ROS production in the mitochondria by regulating GSH content and influenced the intrinsic apoptotic pathway to improve cell survival. We used sh-PICK1 to inhibit PICK1 expression in HK2 to further explore whether PICK1 could protect TECs in septic conditions. Our results demonstrated that PICK1 was upregulated after LPS treatment. Silencing PICK1 increased cell and tissue damage. According to the Hoechst staining, flow cytometry, and western blotting results, knockout of PICK1 raised LPS-induced ROS production and aggravated the apoptosis of renal TECs. We further confirmed that ROS production activated the ASK1-p38MAPK apoptosis pathway and eventually led to cell and tissue damage. Therefore, PICK1 may play an essential role in LPS-induced renal TEC apoptosis. Similarly, immunohistochemistry and pathological sections showed that FSC231 could increase the expression of an apoptotic protein in renal cells and aggravate renal tissue injury. Both in vitro and in vivo experiments showed that silencing PICK1 reduced the production of GSH but increased the ROS in the LPS-induced AKI model. NAC is a kind of ROS scavenger [15, 43]. Pretreatment with NAC significantly decreased apoptosis and ROS and alleviated the PICK1-inhibition-induced injury of HK2 cells. Based on these results, PICK1 silencing upregulated ROS production by decreasing GSH content, [26] which might explain the mechanism of PICK1-mediated cell survival.

In conclusion, we first demonstrated that the highly expressed PICK1 after LPS-induced AKI might be an endogenous protective factor. Simultaneously, the ASK1-p38MAPK pathway might be involved in the promotion of PICK1-deficiency-induced apoptosis. Specifically, the silencing of PICK1 increased the production of ROS and activated the apoptotic pathway, thus aggravating the apoptosis of HK2 cells. Therefore, PICK1 may be a promising preventive target in LPS-induced AKI.

## Data Availability

All supporting data are included within the main article.
