# Erratum to “PICK1 Deficiency Exacerbates Sepsis-Associated Acute Kidney Injury”

**DOI:** 10.1155/2022/9796317

**Published:** 2022-10-14

**Authors:** Qian Dou, Hang Tong, Yichun Yang, Han Zhang, Hua Gan

**Affiliations:** ^1^Department of Nephrology, The First Affiliated Hospital of Chongqing Medical University, Chongqing 400016, China; ^2^Department of Urology, The First Affiliated Hospital of Chongqing Medical University, Chongqing 400016, China; ^3^Department of Gastroenterology, The First Affiliated Hospital of Chongqing Medical University, Chongqing 400016, China

In the article titled “PICK1 Deficiency Exacerbates Sepsis-Associated Acute Kidney Injury” [1], there is an error in Figure 4 that was introduced during the production process. In Figure 4(d), the Western blot is duplicated from Figure 4(e). The correct Figure 4 is shown below:

## Figures and Tables

**Figure 1:**
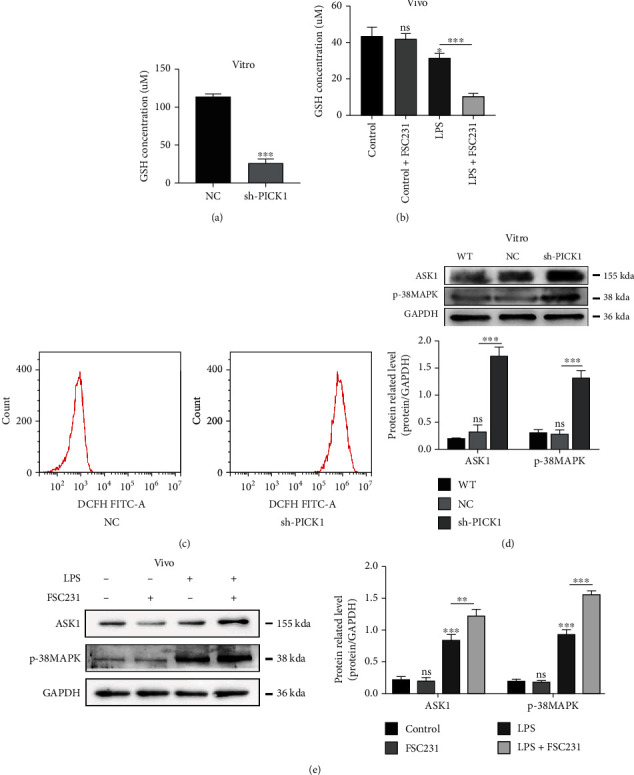
Knockdown of PICK1 increased the production of peroxide and activated the apoptotic pathway after LPS treatment. (a) After treatment with LPS for 24 hours, the content of GSH in the NC and sh-PICK1 groups (NC versus sh-PICK1, ^∗∗∗^*P* < 0.001). (b) The content of GSH in each group of mouse model (LPS versus LPS+FSC231, ^∗∗∗^*P* < 0.001). (c) Representative fluorescence images of ROS. (d, e) Representative Western images of the ASK1 and p38MAPK pathways (NC versus sh-PICK1, ^∗∗^*P* < 0.01. ASK1: control versus LPS, ^∗∗∗^*P* < 0.001. LPS versus LPS+FSC231, ^∗∗^*P* < 0.01. p38MAPK: control versus LPS, ^∗∗∗^*P* < 0.001. LPS versus LPS+FSC231, ^∗∗∗^*P* < 0.001).
